# Concussed patients with visually induced dizziness exhibit increased ocular torsion and vertical vergence during optokinetic gaze-stabilization

**DOI:** 10.1038/s41598-023-30668-y

**Published:** 2023-03-06

**Authors:** Tobias Wibble, D. Frattini, M. Benassi, R. Bolzani, T. Pansell

**Affiliations:** 1 Division of Eye and Vision, Department of Clinical Neuroscience, Marianne Bernadotte Centrum, Karolinska Institutet, Stockholm, Sweden; 2 St Erik Eye Hospital, Stockholm, Sweden; 3 Department of Psychology, University of Bologna, Bologna, Italy

**Keywords:** Predictive markers, Brain injuries

## Abstract

Visually Induced Dizziness (VID) is a common post-concussion sequalae that remains poorly understood and difficult to quantify. The present study aims to identify biomarkers for VID in the form of gaze-stabilizing eye movements. Nine patients with post-commotio VID and nine age-matched healthy controls were recruited by physiotherapists at a local neurorehabilitation centre. Torsional and vergence eye movements were recorded while participants viewed a series of optokinetic rotations where the central- and peripheral regions moved coherently, incoherently, or semi-randomly. Results showed that vergence and torsional velocities were increased in VID patients, reflecting increased oculomotor gain to visual motion, and that responses correlated with symptom severity. Coherent stimulation produced fastest torsional slow-phases across all participants; when faced with confliction directional information, eye movements tended to follow the direction of the central visual field, albeit at slower velocities than during coherent motion, meaning that while torsion was sensitive to visual content of the entire visual field it expressed directional preference to the central stimulation. In conclusion, post-commotio VID was associated with faster slow-phases during optokinetic gaze-stabilization, with both vergence and torsion being correlated to symptom intensity. As torsional tracking remains inaccessible using commercial eye-trackers, vertical vergence may prove particularly accessible for clinical utility.

## Introduction

Post-concussion syndrome (PCS) encapsulates a wide range of symptomologies, from headaches to dizziness and visual disturbances^1^. Visual motion hypersensitivity represents one of the most common sequalae, affecting 70–80% of those affected by blunt head injuries^2^. The condition, also known as Visually Induced Dizziness (VID), is believed to be caused by a reweighing of sensory inputs after which the patient becomes more visually dependant^3^. VID patients have traditionally been viewed as vestibular, due largely to the nature of their symptoms being described as dizziness. Still, studies have failed to identify any correlation between vestibular testing and dizziness caused by visually dependency^4^. It may therefore be argued that Visually Induced Dizziness be treated as a form of non-vestibular vertigo, i.e. a condition associated with typically vestibular symptoms albeit in the absence of central or peripheral disorders. Vertigo as a whole constitutes a severe burden on health-care services^5^, and non-vestibular vertigo has been reported to be as high as 40% of vertiginous patients^6^. It has been argued that the biggest obstacle in identifying these patients lies in the absence of objective clinical tests^4^.

The vestibular/oculomotor screening (VOMS) assessment has proven a robust diagnostic tool, as well as a predictor of recovery times for VID in PCS^7,8^. This includes testing the vestibulo-ocular reflex (VOR), which tests the integrity of the vestibular system’s capacity to reflexively redirect the eyes to compensate for head movements. The optokinetic response (OKR) which allows the eyes to reflexively pursue a moving visual scene has also been affected in concussed patients, who express prolonged optokinetic after-nystagmus (OKAN)^9^. An increased OKAN reflects a heightened build-up of the velocity storage mechanism at the level of the vestibular nuclei, which incorporates both visual and vestibular motion information^10^. Considering the combination of visual and vestibular symptoms in PCS patients, furthering our understanding of how gaze-stabilization may be altered in concussed individuals could prove to be of clinical and academic significance.

The brain’s ability for correlating visual, vestibular, and somatosensory information is critical for maintaining postural control and proper motion perception^11,12^. It has been theorized that a key mechanism of injury in PCS is through diffuse axonal injuries (DAI)^13^, and several imaging studies have shown how several neural connections relaying vision information have been functionally altered in VID patients^14–16^. Considering the number of neural structures involved in visual processing^17^, it appears likely that DAI may disrupt the integration of visual motion information at several nodes in the brain. While eye-movement control involves a number of neural structures, the gaze-stabilizing motor commands are ultimately carried out by the brainstem through activity in the vestibular and extraocular motor nuclei^18^. The OKR consequently not only reflects the central integration of visual motion, but converges with the vestibular system on the most fundamental level. For this reason, the gaze-stabilizing motor commands of the OKR may hold significant clinical utility when assessing VID and PCS.

Gaze-stabilizing eye movements common for both the OKR and VOR are ocular torsion (OT) and vertical vergence (VV)^19,20^ both of which are seen during head rolls and when viewing a visual rotation. One may note that vertical vergence has traditionally been viewed as a solely vestibular phenomenon, or as secondary to torsion^21,22^. However, we have in a series of studies shown how OT is sensitive to changes in visual information density, i.e. clutter, and VV to altered motion parameters such as acceleration^23,24^. There is also evidence for VV being suppressed by visual input even when induced through optokinetic means, and likely represents a visual activation of the vestibular nuclei^25^. Such a relationship may therefore be of particular interest when aiming to assess patients with VID who express vestibular symptoms from visual motion.

This study consequently aims to identify biomarkers for visually induced dizziness in post-concussion syndrome. Participants viewed a range of visual rotations while their optokinetic eye-movement responses of ocular torsion and vertical vergence were tracked. These responses were then correlated with symptom severity. We hypothesise that VID patients will express stronger VV eye movements compared to healthy controls, as reflective of a greater engagement of vestibular neural structures from visual motion.

## Material and methods

### Participants

Nine concussed patients with visually induced dizziness (VID) (7 m, 2f.; age 36.0 ± 11.2) and nine healthy controls (7 m, 2f.; age 39.7 ± 10.1) were recruited for the study. Patients were recruited through a local physiotherapy clinic specializing in neurological rehabilitation. Patients complaining of visually induced dizziness following concussion were informed of the study, and upon expressing an interest in being included were contacted by the research group to plan for their participation. The presence of VID was evaluated by the participating clinic, with the main indicator of inclusion being debilitating dizziness in visually cluttered surroundings, such as supermarkets, scrolling on the phone, watching TV, or traveling on transportation through areas with visually cluttered peripheries. One patient was excluded for lacking binocular vision, and one was excluded due to a pre-existing neurological condition.

All subjects exhibited normal corrected visual acuity (VA; ≥ 1.0), stereoscopic vision (TNO ≤ 60 arc seconds), normal vestibular ocular reflex as tested by the head-impulse test, as well as normal eye motility. Further vestibular criteria were set to exclude any history of vestibular pathologies or dysfunctions. All nine patients had been diagnosed with a traumatic brain injury (TBI) due to impact accidents as indicated by a clinical evaluation in a hospital setting. Average duration from symptom-provoking concussion to inclusion was 31.44 ± 61.04 months (mean ± standard deviation), reflecting the clinical population seeking healthcare at the participating clinic. Eight patients had mild TBI, while one participant had suffered a moderate TBI as indicated by small cerebral bleedings. To minimize the risk of patient drop-out due to the provocative nature of the visual stimulation only patients with a moderate level of VID were invited, as indicated by an assessment of a clinical specialist familiar with the procedure. The Visual Vertigo Analog Scale (VVAS) and Dizziness Handicap Inventory (DHI) were submitted to evaluate the subjective severity of respectively visual vertigo and vestibular symptoms. The research protocol adhered to the Declaration of Helsinki. All participants received written and oral descriptions regarding the nature of the study and provided informed consent at the time of recruitment. The ethical permit was approved by the Regional Ethics Committee of Stockholm (EPN 2018-1768-31-1).

### Procedure

Participants were exposed to rotational optokinetic stimulations in a dimly illuminated room at a fixed luminosity (34 lx). The subject's head was positioned on a height-regulated chinrest at 60 cm eye-screen distance (Sharp LCD 55", 50 Hz, Sharp Electronics, Hamburg, Germany) so that subjects’ gaze would be levelled on a central fixation point, on which subjects were instructed to maintain their gaze for the duration of the protocol. The torsional optokinetic reflex and the vertical vergence response were recorded using a video head-mounted binocular eye-tracker recording at a sampling rate of 100 Hz (Chronos Eye Tracker; Chronos Inc., Berlin). Eye-movement data was collected in terms of mean, and peak slow-phase velocities, as well as torsional nystagmus beats frequency, which were extracted from the ocular torsion and vertical vergence responses as observed during each stimulation phase. The data was retrieved and subsequently analysed according to established and previously published procedures^23,24^, where each torsional slow-phase velocity was retrieved by divided the difference in starting and ending position of a slow-phase by time; all slow-phases were identified and calculated manually. Vertical vergence responses were retrieved by subtracting the position of the right pupil from the position of the left, and were collected over the same time period as each corresponding torsional slow-phase. Both ocular torsion and vertical vergence velocities are expressed in absolute values, where mean velocities indicate the average velocity of all the slow-phases retrieved during a single optokinetic stimulation, whereas peak velocities refers to the fastest slow-phase velocity retrieved during a single trial. The torsional nystagmus beats were retrieved from the eye movement plots. Stimulation trials in which every ocular torsion slow-phase were separated by a blink were excluded from the nystagmus frequency analysis, leading to an inclusion of 137 trials out of 144 in the statistical analysis.

Furthermore, the general direction of each ocular torsion (i.e. clockwise or counter-clockwise) and vertical vergence were retrieved for each stimulation period. Slow-phase directions were categorized as being entrained by the central or peripheral motion direction whenever 85% of all the slow-phases showed the same directionality. Whenever the percentage was lower than 85%, the directionality was categorized as mixed, indicating that ocular torsion and vergence lacked any clear directional preference; this was done on the basis that each optokinetic stimulation generated on average seven torsional slow-phases, allowing on average one to be in the opposite direction before that trial was considered mixed. For reference, clockwise ocular torsion and negative vertical vergence are generated by clockwise movement of the optokinetic stimulation whereas the opposites are generated by a counter-clockwise optokinetic stimulation.

### Optokinetic stimulation

**Figure 1:**
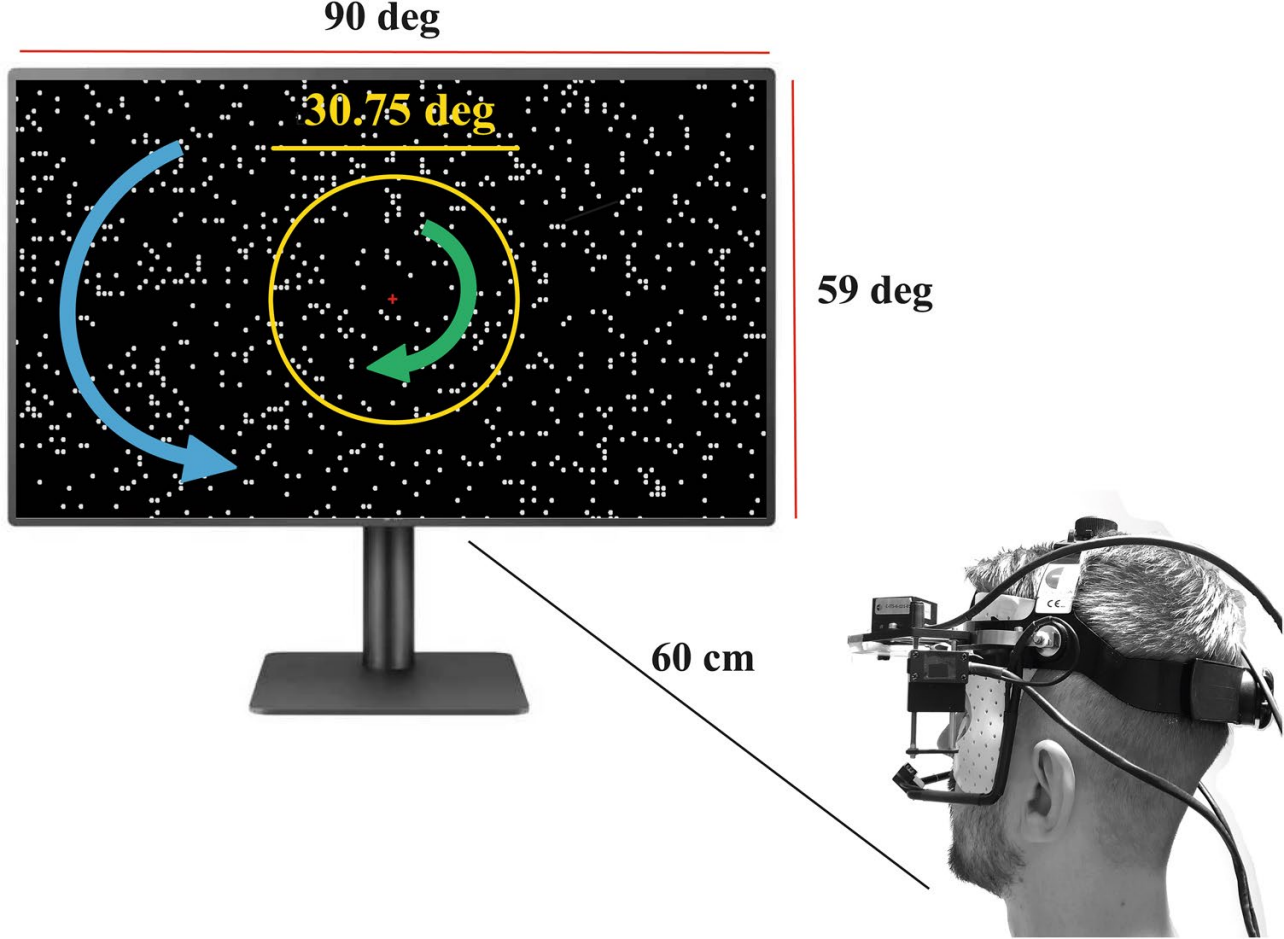
An illustration of the visual scene (left) as viewed by a participant wearing the Chronos Eye Tracker at an eye-screen distance of 60 cm (right). The visual scene was divided into a central area (yellow circle) and a peripheral area that was delineated by the edges of the monitor (red lines). The central area encompassed a diameter of 30.75° of the participant’s visual field, while the peripheral area extending 90° horizontally and 59° vertically. The motion pattern outlined in this example represents a *non-coherent* stimulation consisting of clockwise central motion and counter-clockwise peripheral motion. The central red cross acted as the fixation point on which participants were asked to fix their gaze, while maintaining the chin on a chinrest, for the duration of the stimulation protocol.

**Figure 2:**
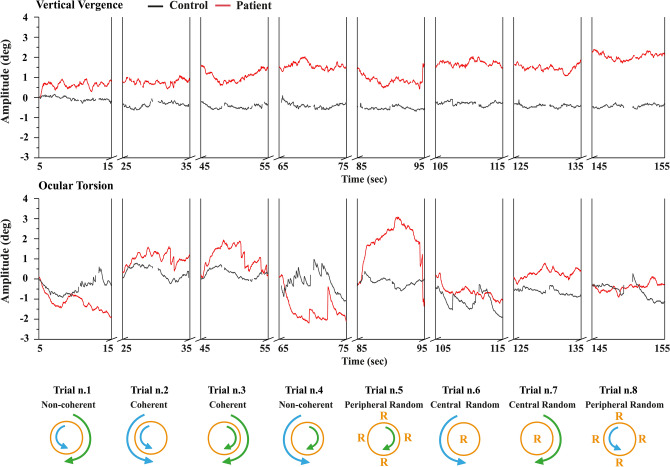
Stimulation pattern effect in patients and controls on ocular torsion and vertical vergence responses. Graphical illustration of the vertical vergence optokinetic response recording (above), and the ocular torsion response (below) between a healthy individual (Black) and a concussed patient with VID (Red). Baseline values, i.e. eye position at rest, has been removed from the figure for fitting purposes as indicated by breaks in the X-axis. Below is a graphical illustration of all stimulation patterns, numbered according to the order they were presented at during the session and corresponding with the eye movements outlined directly above.

The optokinetic stimulation consisted of a matrix of white dots moving at a constant angular velocity of 36.4 /s on a black background. Each dot subtended a visual angle of 0.66°, and the optokinetic stimulation covered a visual angle of 90° horizontally and 59° vertically. The optokinetic stimulation was segmented into a central circular area, subtending 30.75° of the visual field, and the mid-peripheral area reached up to 90° of the visual field horizontally (Fig. 1). Stimulations differed in movement coherence between central and mid-peripheral visual fields, rotating either clockwise, counter-clockwise, or in random directions. The combination of movement directions and coherences yielded eight different optokinetic patterns. These patterns were grouped in four categories: *coherent* when both central and peripheral fields moved in the same direction, *non-coherent* when they moved in opposite directions, *central-random* when Brownian motion was displayed centrally, and *peripheral-random* when Brownian motion was displayed peripherally. Examples of all motion patterns can be found in Fig. 2 together with the recorded eye movements from one participant during all stimuli, the order of which were balanced between subjects.

Each session started with subjects viewing a static image of the dots for five seconds during which baseline eye positions were collected; these baseline values reflect the stable period one second before each trial. Each optokinetic pattern was then presented for a duration of 10 s, here referred to as a trial. This was followed by 10 s resting period during which the scene was frozen. Three different sessions were designed, between which the order of presentation of the respective optokinetic patterns had been shuffled. This was done to mitigate habituation effects from any one specific motion pattern while maintaining patient comfort by limiting the testing period. Each subject was exposed to one session of 8 trials, lasting 160 s.

### Statistical analysis

Statistical analysis was performed using IBM SPSS Statistics 26 (IBM, Armonk, NY, USA). A Shapiro Wilk test revealed that mean torsional, peak torsional, mean vergence and peak vergence optokinetic response values were not normally distributed; the Levene test of equal variance indicated a lack of homoscendasticity of all dependent variables. For this reason, a Generalized Linear Mixed Model (GLMM) with full factorial design was adopted for each dependent variable. Fixed between-subject predictors included group (VID patient or healthy control), whereas the stimulation pattern (i.e. *coherent*, *non-coherent*, *central-random or peripheral random*) and central-field optokinetic motion direction (i.e. clockwise or counter-clockwise) were included as repeated measure factors. Lastly, trial sequence was included as a random effect. A simple contrast, with Sequential Sidak adjustment for multiple comparison on the alpha level, was applied to stimulation pattern factor levels using the optokinetic pattern *coherent* as reference category. A pairwise contrast, with Sequential Sidak adjustment for multiple comparison on the alpha level, was applied to each interaction effect to examine the influence of each optokinetic pattern stimulation and direction of motion on the eye movement responses between groups. The sample size for GLMM test was evaluated using the R language simPower procedure and using a sample of nine subjects per group yielded a power of 80.4% for main effects.

A frequency analysis with Pearson Chi^2^ was applied to evaluate the association between patients and controls with reference to ocular torsion and vertical vergence direction during each stimulation pattern. Furthermore, the ocular torsion to vertical vergence ratio in terms of velocity and directionality of each slow-phases recorded during the totality of the stimulation session between the two groups was investigated through a Spearman’s rank correlation test.

The correlation between VVAS- and DHI scores with the optokinetic responses was analysed through Spearman’s rank correlation test. A two-tailed significance alpha level of 0.05 was set for all the statistical test executed in the analysis.

## Results

None of the participants dropped off due to excessive symptoms during the study. One VID patient was unable to keep their eyes open during the central-random trial. Therefore, a total of 71 optokinetic response measurements were collected for the VID group as compared to 72 in the control group.

### Differences in gaze-stabilization between patients and controls

**Figure 3:**
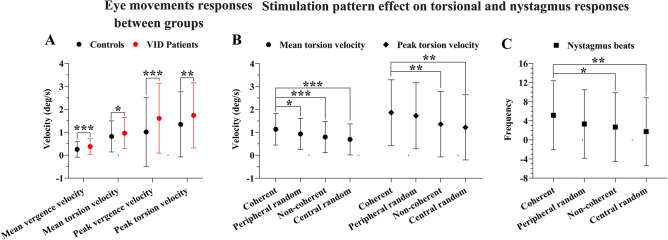
(**A**) An interval plot illustrating the optokinetic slow-phase responses velocities as absolute values (degrees per second), and the 95% confidence interval (error bars), between healthy individuals and concussed individuals with VID. (**B**) Interval plot illustrating the modulating effect of different optokinetic stimulation patterns over the mean torsion and peak torsion optokinetic slow-phase velocities (degrees per second), and the 95% confidence interval (error bars). (**C**) Interval plot illustrating the modulating effect of different optokinetic stimulation patterns over the number of nystagmus (frequency), and the 95% confidence interval (error bars). *p < .05; **p < .01; ***p < .001.

Concussed patients exhibited faster optokinetic eye movements compared to controls independently of the optokinetic pattern, motion direction and trial sequence. This was manifest across all gaze-stabilizing slow-phases (see Fig. 3A). Mean torsional velocities were significantly different in patients compared to controls (F (1,99) = 5.488, *p* = .021;). A similar response pattern was observed for peak torsional slow-phase velocities, as patients expressed faster slow-phase velocities compared to controls (F (1,101) = 9.851, *p* = .002). Patients also had significantly faster vergence-slow-phases, both in terms of mean (F (1,109) = 17.838, *p* < .001), and peak (F (1,110) = 21.276, *p* < .001) responses. No significant difference was found between clockwise and counter-clockwise stimulus movement.

**Figure 4:**
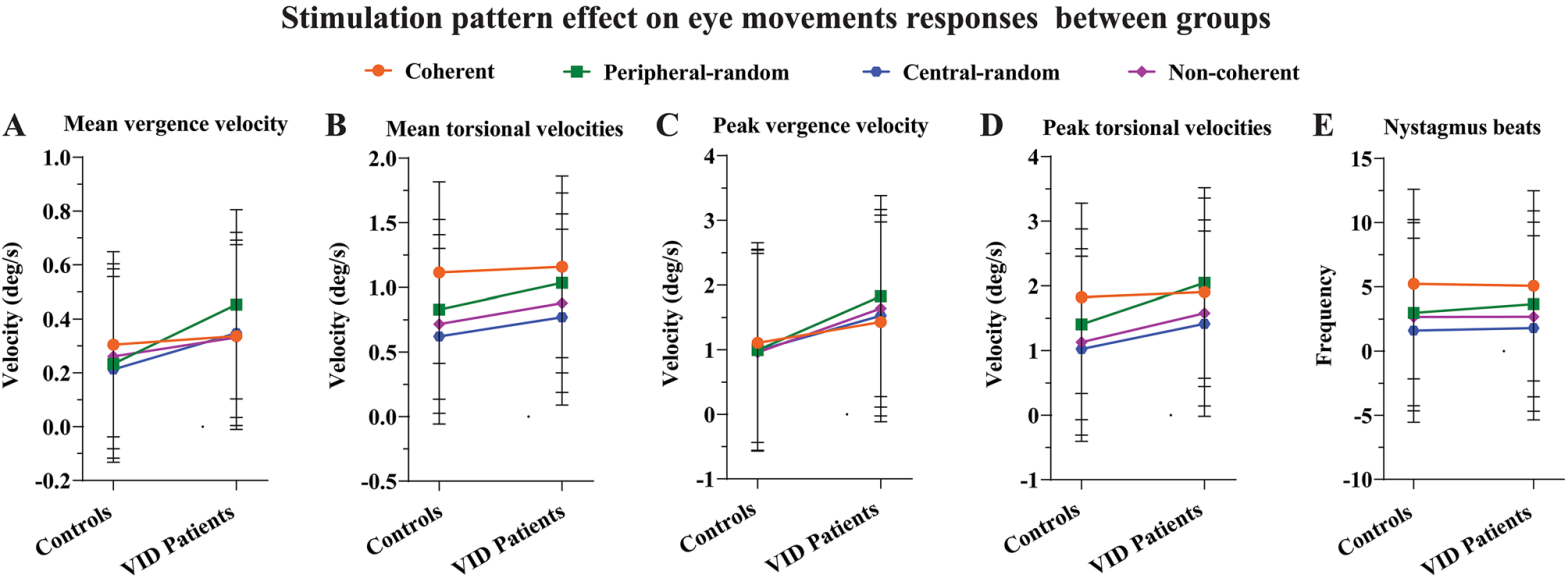
(**A**) Interval plot illustrating the modulating effect of different optokinetic stimulation patterns over the mean vergence slow-phase velocities (**A**), mean torsional velocities (**B**), peak vergence velocities (**C**), and peak torsional velocities (**D**) in degrees per second, as well as the number of nystagmus beats (**E**), illustrated with the 95% confidence interval (error bars) between healthy individuals and concussed individuals with VID.

The stimulation pattern produced significantly different mean torsional velocities (F (3,54) = 10.851, *p* < .001; see Fig. 3B), which showed that the fastest responses were observed during *coherent* optokinetic patterns for all participants compared to *peripheral-random* (t (60) = 2.091, *p* = .041) and *non-coherent* (t (54) = 3.653, *p* < .001) trials. The slowest responses were found during the *central-random* stimulation motion patterns (t (47) = 5.348, *p* < .001). The stimulation-pattern produced a significant effect also on peak torsional slow-phase velocities (F (3,54) = 6.584, *p* = .001) which showed that *coherent* stimulation patterns generated a significant heightened peak activity compared to *non-coherent* (t (55) = 2.988, *p* = .008) and *central-random* stimulation patterns (t (53) = 3.968, *p* = .001) for all participants. While the interaction motion patterns by groups was not significant, there were clear trends for specific conditions (see Fig. 4).

The nystagmus response was also significantly affected by the stimulation pattern (F (3,48) = 5.806, *p* = .002; see Fig. 3C) with *coherent* stimulation trials generating the highest frequency of nystagmus beats. A significant decrement, compare to *coherent* stimulation patterns, was observed in *non-coherent* (t (53) = 2.390, *p* = .040) and *central-random* (t (45) = 3.963, *p* = .001) trials. There was no difference between groups in terms of nystagmus frequencies.

Altogether these findings means that concussed patients with VID expressed faster gaze-stabilizing slow-phase velocities compared to healthy controls. It also means that the motion-pattern effect recorded in the study population tended to be exacerbated in the patient group for all stimulation patterns. While this phenomenon did not reach statistical significance, one may note from Fig. 3B that the relative difference in slow-phase velocity between patients and controls was increased during the *peripheral-random* condition for all eye movements.

**Figure 5:**
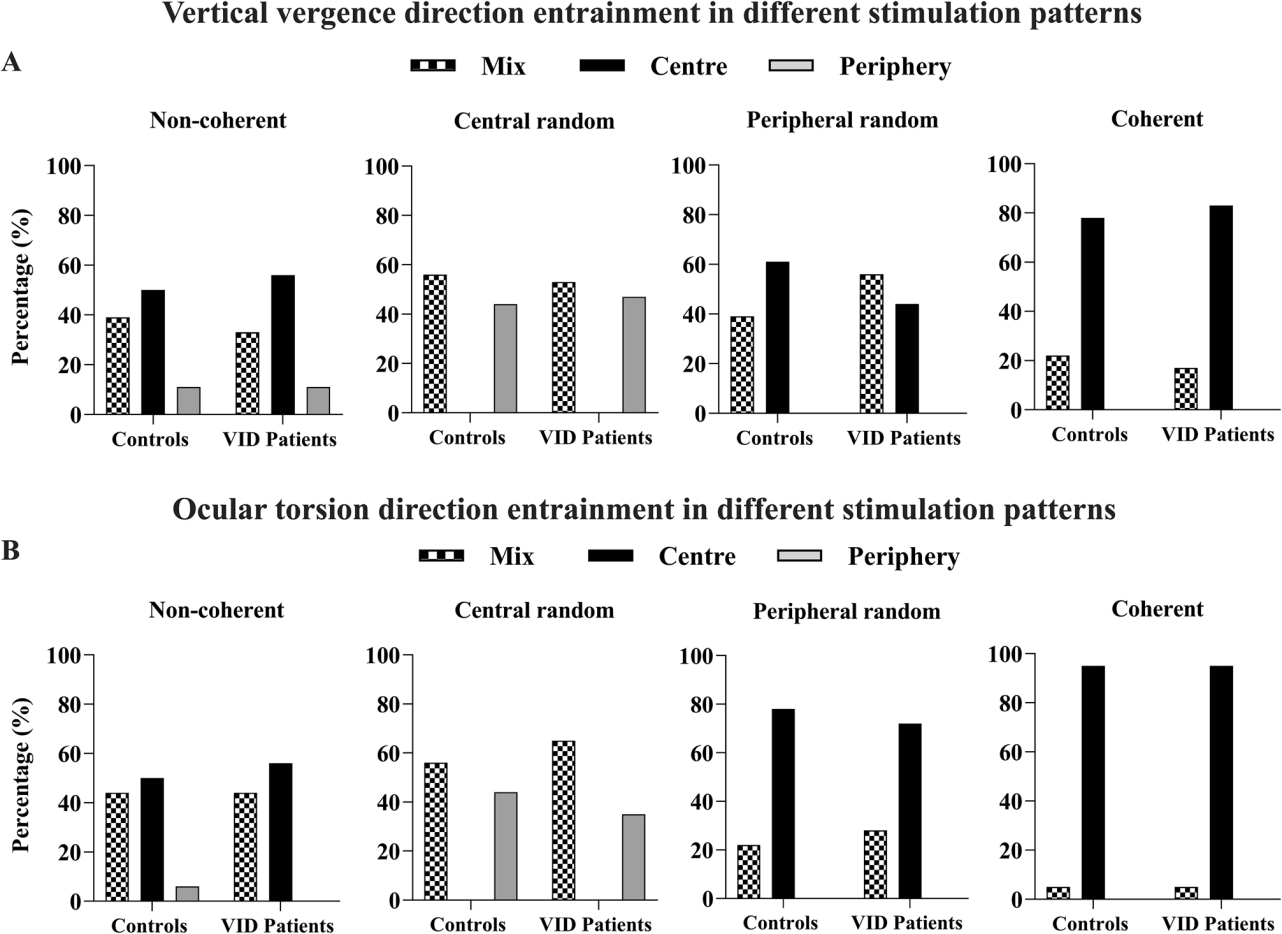
(**A**) Histogram plot illustrating the percentage of vertical vergence direction entrained by the central or peripheral portion of the different optokinetic patterns between groups. (**B**) Histogram plot illustrating the percentage of ocular torsion direction entrained by the central or peripheral portion of the different optokinetic patterns between groups.

The present study also investigated the direction of each eye movement in relation to the optokinetic direction. Frequency analysis did not reveal any significant difference between groups concerning the torsional and vertical vergence direction entrainment relative to optokinetic patterns (see Fig. 5). Generally, the direction of the optokinetic response followed that of the central field; when the central field presented random motion, the majority of trials exhibited no clear directional preference.

Concerning the relationship between torsion and vergence, torsional slow-phases showed a significant negative correlation with corresponding vertical vergence movements (Rho (942) =  − .48, *p* < .001). Considering the two experimental groups separately, the VID groups presented a lower negative correlation between ocular torsion and vergence slow-phases velocity (Rho (508) =  − .43, *p* < .001) than the control group (Rho (432) =  − .55, *p* < .001). Altogether, these findings show that torsion and vergence behaved in a predictive way relative to each other, and that the ratio between the two eye movements was less strict in the patient group.

### Symptom severity

Symptom’s severity questionnaire significantly correlated with the optokinetic responses to a great extent as indicated by the Spearman rho correlation analysis. In healthy participants, higher dizziness symptoms’ scores evaluated through the Dizziness Handicap Inventory (DHI) positively correlated with all the optokinetic variables. The severity of visual vertigo symptoms was measured through the Visual Vertigo Analog Scale (VVAS) and DHI scores. Both showed a significant positive correlation with VID individuals' optokinetic responses (see Table 1).

**Table 1:** Correlation table between slow-phase velocities for each eye movement and the Visual Vertigo Analogue Scale (VVAS) and Dizziness Handicap Inventory (DHI) for both healthy participants and patients suffering from visually induced dizziness (VID).

Symptom severity and eye movement responses correlation table								
Optokinetic responses	Controls				VID patients			
	VVAS		DHI		VVAS		DHI	
	Rho	*p*	Rho	*p*	Rho	*p*	Rho	*p*
Mean torsion	− 0.123	0.301	0.308	0.008	0.266	0.025	0.310	0.009
Peak torsion	− 0.156	0.191	0.308	0.008	0.304	0.010	.0404	< .001
Mean vergence	0.093	0.435	.0569	< .001	0.423	< .001	0.430	< .001
Peak vergence	− 0.272	0.021	0.465	< .001	0.333	0.005	0.289	0.015

## Discussion

This study aimed to identify biomarkers for Visually Induced Dizziness, presenting quantifiable values that can be used in clinical care. Both torsional and vergence eye movements were significantly altered in VID patients, exhibiting increased velocities and poorer adaptation over time. These changes were also linked to symptom severity, as indicated by the VVAS and DHI.

It has been documented that concussed patients with visual dysfunctions are more sensitive to motion in the visual periphery^26^. One may therefore expect that patients would be particularly influenced by rotating visual motion, as a rotating visual field will always cause a greater displacement in the peripheral retina compared to the central region. The torsional velocities recorded in this study therefore fits well within the theoretical framework for how VID patients integrate visual motion; while stimulation pattern did not produce a significant difference between patients and controls, the trend seen in Fig. 3B that random motion in the visual periphery produced relatively greater differences between patients and controls would fit well within the framework of previous findings, showing that concussions have aggravated effects on light detection in the visual periphery compared to the central visual field, as indicated by a prolonged reaction time to light detection^26^. This may be contrasted to the main effect concerning stimulation pattern, where all participants exhibited the fastest torsional slow-phases when viewing coherent optokinetic motion. It would therefore appear that torsional slow-phase velocities are primarily affected by the central visual field. However, the fact that non-coherent stimulation yielded comparatively slow velocities indicate that it is not the relative motion of the central field that dictates the torsional response, but rather a whole-field synthesis of motion with preference to the central region. While patients followed this trend, it appears that they were more readily influenced by peripheral motion.

We have previously shown that ocular torsion is influenced by visual content, as increased visual density information is associated with greater torsional gain in the roll plane^27^. It is well-documented that VID patients experience vertiginous symptoms in visually cluttered surroundings, earning the condition its moniker “supermarket syndrome”^28^. In this context, one may suggest that the increased torsion be indicative of hypersensitive central integration of visual content, as reflected in patients’ reflexive gaze-stabilization. A limitation to this interpretation is the level of attention subjects may pay to the stimuli; we have recently shown that torsional slow-phase velocities reflect a viewer’s alertness level^29^. In this regard, it may be difficult to separate if the increased OT gain is due to increased alertness in VID patients, which may have been caused by their symptoms, or through neurophysiological changes influencing gaze-stabilization directly.

Concerning the direction of torsion, there was no difference between patients and controls, and it generally followed the central field which is line with previous findings^30^, and as torsion and vergence were shown to be correlated to each other across all recordings one may conclude that eye movements were appropriately matched in terms of directionality, and both can be concluded to express a central field preference. In the present study, the introduction of peripheral-random stimulation decreased this directional preference, and conflicting, non-coherent, stimulation reduced it further. It would consequently appear that the central preference is retained, albeit negatively modified by motion in the visual periphery.

It may be noted that both vertical and torsional eye movements stem from the same neural gaze-centre in the riMLF^31^, as compared to horizontal ocular convergence which rely on a network of nuclei, most notably near-response cells near the oculomotor and abducens nuclei^32^, and conjugated horizontal eye movements stemming from an activation of the gaze-centre of the PPRF^33^. With both horizontal and vertical vergence eye movements disrupted in VID patients, it would consequently appear likely that the altered responses stem from changes in efferent neural areas with respect to these structures and gaze-centres, possibly reflecting impairments to network communications. One may note that fixational instability has been described in concussed patients^34^. It is consequently quite possible that the hypothesised mechanism may be allowed by a reduced capacity for visual fixation in these patients, whereas healthy individuals may be capable of visually suppressing the optokinetic responses.

Compared to its torsional counterpart, causes for an increase in vertical vergence gain are less clear. We have recently shown that ocular torsion and vertical vergence may be affected independently of one another, where vergence is more readily influenced by optokinetic motion^23^. Unlike vertical vergence, horizontal vergence, allowing for the eyes to converge or diverge to achieve binocular fusion, has been readily studied in the context of concussion and vertigo^35^. Vergence dysfunction in the horizontal axis has been well-documented in concussed patients and is believed to be caused by a general processing delay of afferent signals, causing decreased convergence and divergence velocities due to decreased signal input to key cortical and subcortical structures^36^. It is also known that voluntary convergence in the horizontal plane is impaired in patients with VID symptoms and post-concussion syndrome^37^. From a subjective perspective, it has been shown that patients with convergence insufficiency experience a higher burden of symptoms, particularly in terms of learning and memory capacity^38^. In more severe cases of concussion, convergence insufficiency has been associated with poorer outcomes in terms of rehabilitation and lasting cognitive problems^39^. It is noteworthy that these studies generally indicate decreased velocities in addition to decreased neural activity in several key neural structures, supporting the delayed-processing theory^40^. One may also note that studies in children with vertigo have shown that while these patients may have intact vestibular functioning, they express vergence abnormalities^41^. The ruling theory is that it is the vergence insufficiency that causes symptoms of vertigo, as it can be strongly correlated to the sensation and mitigated by orthoptic training^42^. It should be noted that while these children struggle to adjust to different viewing distances, vergence velocities have been shown to be comparable to those of healthy control^42,43^. These findings can be contrasted to increased vertical vergence velocity observed in the present study. Unlike horizontal vergence, one may suggest that vertical vergence has very little physiological function in the absence of visual disparities^44^; a previous study has shown that antithetically to horizontal vergence, binocularity appears to depress vertical vergence^25^. The question remains whether this increased vertical divergence may contribute towards symptoms of vertigo, or if it acts as an outlet for a central pathological misprocessing. Considering the low gain observed is it tempting to suggest that the latter may be the case, though future studies employing real-life scenario and visual motion is warranted. Currently, these findings may best be viewed as outcomes for this testing procedure, aimed at producing objective and clinical biomarkers, and not representative of the physiological response to real-world motion in three dimensions. In addition, one further limitation of the present study was that horizontal vergence was not clinically evaluated as part of the recruitment process; eye movement data was however visually inspected in the horizontal plane, to ascertain visual fixation, but no statistical comparisons were performed relating to horizontal vergence at baseline. It was consequently not possible to determine if there is a connection between horizontal vergence insufficiency and the recorded increase in vertical vergence gain.

From a neurophysiological perspective, it has previously been put forward that dissociated vertical divergence (DVD), a condition seen in children who due to amblyopia express vertical divergence and a perceptual tilt of the viewed scene, is an expression of an otherwise vestigial dorsal light reflex^45,46^. Considering the reliance on vestibular input towards the dorsal light reflex^47^, and the association of vestibular signalling as a cause for vertical vergence^21^, we put forward that the current study supports the notion that visually induced vertical divergence may reflect an optokinetic activation of the vestibular system^25^. An established method of estimating the optokinetic activation of the vestibular system is through quantifying nystagmus frequency, as it may be seen as a reflection of the velocity storage mechanism (VSM), where an increased frequency of beats reflects greater motion integration into the vestibular system^48^. In this context, the fact that patients and controls did not differ in terms of nystagmus beat frequency could arguably suggest that there is no significant increase in visual motion sensitivity towards the VSM in concussed patients with VID which is in contrast with the increased oculomotor velocities noted in the patient population. It should be noted that the time constant for the VSM allows it to be best observed during rotations longer than 10 seconds^49^, and as the stimulation period in the current study equalled this duration, nystagmus frequency may be considered inadequate for drawing conclusions relating to the VSM as an indicator for how the optokinetic stimulation may have affected the vestibular system. Furthermore, seven trials had to be removed due to excess blinking during the stimulation period. Out of these seven trials, six belonged to patients and one to the control group; this was likely due to subjective discomfort during the protocol. Considering these factors and the great between-subject variability, it may be difficult to draw conclusions based on nystagmus frequency in the present study, and future studies may benefit from a more prolonged stimulation period.

This study aims to identify possible oculomotor biomarkers for VID in concussion, and as such it is of interest to know if these eye movements may be correlated with subjective symptoms. The VVAS and DHI aim to measure the subjective sensation of dizziness or vertigo. This study found that both VV and OT were positively correlated with VVAS and DHI in patients, while similar correlations were only noted for DHI in healthy participants. As the VVAS is dedicated to VID patients this may have been expected. It is nevertheless notable that eye-movement parameters reflect these subjective assessments. It further highlights the value of using gaze-stabilizing eye movements as biomarkers for VID and supports the credibility of both DHI and VVAS questionnaires.

In conclusion, this study found that both ocular torsion and vertical vergence exhibited increased slow-phase velocities in the patient group, and we therefore suggest that both these gaze-stabilizing eye movements may serve as potential biomarkers for Visually Induced Dizziness in patients with post-concussion syndrome. Both eye movement responses were also correlated with subjective complaints relating to dizziness as indicated by the DHI and VVAS. As pupil position is easier to measure using commercial eye-trackers, we suggest that vertical vergence velocities may hold clinical utility in assessing patients with VID. As ocular torsion and vertical vergence can be used to infer the central integration of both optokinetic stimulation and self-motion, the present study finds that they may serve as general visuo-vestibular biomarkers for motion processing in a clinical setting.

## Data Availability

The datasets used and/or analysed during the current study available from the corresponding author on reasonable request.
